# Autonomic Nervous System Activity in Young Subjects Exposed to Orthostatic Posture and Emotional Visual Stimuli: A Pilot Study

**DOI:** 10.3390/biology15030266

**Published:** 2026-02-02

**Authors:** Sandica Bucurica, Ioana Toader, Constantin Pistol, Ionela Maniu, Ilinca Savulescu-Fiedler

**Affiliations:** 1 Department of Gastroenterology, “Carol Davila” University of Medicine and Pharmacy, 050474 Bucharest, Romania; sandica.bucurica@umfcd.ro; 2 Department of Gastroenterology, University Emergency Central Military Hospital “Dr. Carol Davila”, 010825 Bucharest, Romania; 3 Department of Internal Medicine, “Carol Davila” University of Medicine and Pharmacy, 050474 Bucharest, Romania; ilinca.savulescu@umfcd.ro; 4 Department of Internal Medicine, Coltea Clinical Hospital, 030167 Bucharest, Romania; 5 Physics Department, University of Bucharest, 405 Atomistilor Street, 077125 Ilfov, Romania; costi.pistol@fizica.unibuc.ro; 6 Department of Mathematics and Informatics, Faculty of Sciences, Lucian Blaga University of Sibiu, 550012 Sibiu, Romania; 7 Research Team, Pediatric Clinical Hospital Sibiu, 550166 Sibiu, Romania

**Keywords:** autonomic nervous system, heart rate variability, emotion, postural changes, autonomic adaptability

## Abstract

Heart rate variability (HRV) reflects autonomic nervous system (ANS) activity and provides insight into physiological and emotional regulation. Evaluating HRV during postural and emotional challenges may help characterize autonomic adaptability in healthy individuals. HRV was recorded in 24 young medical residents (17 females, 7 males; mean age 27.04 ± 1.97 years) during four conditions: rest, orthostatic standing, and exposure to positive and negative emotional images. Each session lasted five minutes. Anxiety and depression were assessed using the Hospital Anxiety and Depression Scale. Heart rate increased significantly only during standing, consistent with sympathetic activation with postural change. Spectral and normalized HRV parameters (nLF, nlf, LF/HF, and normalized coherence) were lowest at rest and increased during standing and emotional image exposure, particularly in males. Parasympathetic indices showed opposite trends. Emotional image exposure did not produce significant differences between positive and negative valence at the group level; however, sex- and anxiety-related patterns emerged. Females with anxiety showed increased heart rate during positive image exposure, whereas non-anxious females exhibited higher heart rate responses to negative images. Orthostatic challenge elicited the strongest autonomic response, whereas emotional visual stimuli induced subtler, sex- and anxiety-dependent autonomic modulation without overall changes in heart rate. These preliminary observations suggest that anxiety and sex may be associated with differences in cardiac autonomic regulation in young healthy adults. These results should be interpreted cautiously, given the pilot design, the small sample size (*N* = 24), the imbalance between sexes, the exclusion of the depression subgroup from inferential analyses, and the use of non-validated emotional visual stimuli

## Simple Summary

Heart rate variability reflects the activity of the autonomic nervous system and serves as a physiological marker of emotional responsiveness. It indicates the functional connection between the heart and the central nervous system regions involved in regulating emotion and behavior. According to the heart rhythm coherence hypothesis, vagal afferent signaling contributes to emotional self-regulation, while negative emotions reduce vagally mediated heart rate variability. Large-scale biofeedback data confirmed associations between heart rate variability parameters and self-reported emotional states. In this study, heart rate variability analysis revealed distinct autonomic nervous system responses to orthostatic and emotional challenges in young healthy adults. Heart rate increased only during standing, indicating sympathetic activation, while emotional visual stimuli modulated autonomic balance without altering heart rate. Spectral parameters were lowest at rest and increased significantly during standing and image exposure, particularly in males, whereas parasympathetic indices showed opposite trends. Sex and anxiety appeared to be associated with differences in autonomic responses: anxious females exhibited higher heart rate during positive image exposure, while non-anxious females responded more to negative stimuli. Normalized coherence correlated positively with the low-to-high frequency power ratio across active conditions, supporting their potential role as markers of cardiac–autonomic integration and emotional adaptability.

## 1. Introduction

Cardiac frequency varies dynamically at rest and under different physiological or emotional conditions. Heart rate variability (HRV) reflects the activity of the autonomic nervous system (ANS) and serves as an indicator of the emotional response [1]. HRV mirrors the integrity of the network that functionally links the heart to the regions belonging to the central nervous system (CNS) involved in contextual analysis and behavioral regulation [2].

According to the neurovisceral integration model proposed by Thayer and Lane, ANS responses are modulated by cortico–subcortical interactions that integrate cognitive, affective, and physiological processes [3,4]. Resting HRV level reflects the prefrontal cortex’s (PFC) efficiency in controlling subcortical circuits, generating more flexible and contextually appropriate emotional responses [4,5]. A higher HRV is typically associated with superior emotional regulation and lower anxiety levels, while reduced HRV is observed in individuals with anxiety or depressive symptoms [6,7,8,9,10]. Negative affect, depression, and adverse social experiences have all been linked to diminished HRV, reflecting impaired autonomic control [2,11].

Accumulating evidence supports a close relationship between HRV, cognition, and emotion [12]. Individuals with higher HRV tend to exhibit a pro-social attitude and more empathy [13]. Elevated resting HRV values are associated with more intense PFC activity, better cognitive performance, superior emotional control, and superior memorization performance [2,14,15,16]. The reciprocal is also valid: the more intense the PFC activity, the more the heart rate and HRV can be modulated, which may have therapeutic utility [3].

According to the heart rhythm coherence hypothesis, cardiovascular afferent vagal signaling contributes to cognitive modulation and emotional self-regulation [17]. Some studies underscore that negative emotions are known to reduce vagally mediated HRV [18]. A large-scale retrospective study in the HRV biofeedback field, using the Inner Balance app, compared data from 1.8 million biofeedback sessions between 2019 and 2020 and established a relationship between various parameters of HRV (in both time and frequency domains) and self-reported emotional status [19]. HRV metrics are thus widely used as quantitative indicators of ANS activity [20]. The oscillations in cardiac electrical activity can be decomposed into time- or frequency-domain parameters that describe sympathetic and parasympathetic influences. HRV may be assessed over long-term (24 h), short-term (≥5 min), or ultra-short-term (<5 min) recordings of cardiac electrical activity, depending on the objective of the investigation.

## 2. Materials and Methods

### 2.1. Study Objectives

The primary objective of this study was to assess autonomic nervous system (ANS) activity, as reflected in heart rate variability (HRV), during orthostatic stimulation and while viewing emotionally charged visual stimuli (i.e., positive and negative content). Secondary objectives included evaluating HRV responses according to anxiety and depression scores.

### 2.2. Participants

We conducted a prospective study involving resident doctors of similar age, health status, and academic level. All participants were informed of the study’s theoretical basis, purpose, and protocol, and provided written informed consent before enrollment. Anxiety and depression were evaluated using the Hospital Anxiety and Depression Scale (HADS) [21]. The Hospital Anxiety and Depression Scale (HADS) is a widely used, validated screening instrument with good psychometric properties, with satisfactory reliability and internal consistency, with reported Cronbach’s alpha values typically ranging from 0.80 to 0.93 for the anxiety and depression subscales. In the present study, we used the validated Romanian-language version of HADS, which has been standardized for clinical and research use. The questionnaire was self-administered by all participants.

The sample size was determined by the study’s limited duration and the availability of eligible participants during this period. The study was conducted within a predefined time window, during which all resident doctors who met the inclusion criteria and agreed to participate were consecutively enrolled. Thus, the final sample represents the entire accessible population of eligible residents during the study period. This approach allowed us to minimize selection bias while maintaining a homogeneous cohort in terms of age, health status, and professional workload.

The study was conducted between 28 May 2024 and 1 October 2024, with approval from the institutional ethics committee (No. 6006/10.04.2024). Participants were instructed to avoid alcohol, caffeine, nicotine, chocolate, and energy drinks for 1 h before testing; refrain from smoking 30 min prior; avoid vigorous physical activity the day before; and have had a good night’s sleep [22]. All recordings were performed in the morning.

### 2.3. Experimental Setting

Recordings were conducted in a quiet, closed room maintained at 20–24 °C, with the sole participation of the person designated to perform the recordings. All electronic devices (e.g., watches, phones) were removed during recording time.

### 2.4. Recording Protocol

HRV data were collected using the emWave Pro system (HeartMath Inc., Boulder Creek, CA, USA). Each recording session lasted 5 min, meeting the recommended minimum duration for reliable HRV analysis, for 1 min for SDNN, rMSSD, and HR recordings, 5 min for all spectral bands [23,24,25,26].

Visual stimuli consisted of 25 images with positive and 25 with negative emotional content, displayed on a computer screen for 12 s per image. Before data collection, each participant completed a 10 min rest period in a seated position to promote relaxation (in a comfortable chair with a backrest and arms).

The visual stimuli were selected from publicly available images obtained via Google Image Search. Images were selected by the investigators to represent positive or negative emotional content clearly and were standardized with respect to number, presentation duration, and display conditions across participants. Although a validated emotional image database was not used, all subjects were exposed to the same stimuli, ensuring internal consistency of the experimental protocol. This aspect is acknowledged as a methodological limitation.

Four sessions were performed sequentially, separated by rest intervals, to minimize carryover effects:

− Session A (Rest): Participant seated comfortably without visual or auditory stimuli; spontaneous breathing; HRV recorded for a duration of 5 min.
− Session B (Standing): Participant standing upright; HRV recorded for 5 min.
− Session C (Positive images): Participant seated; viewed 25 positive images for 5 min.
− Session D (Negative images): Participant seated; viewed 25 negative images for 5 min.

Rest intervals, without HRV recording, were as follows:

− Between session B and session C: the subject returned to the sitting position for 10 min, no stimuli.
− Between session A and session B, as well as between session C and session D: the subject returned to the sitting position for 5 min, no stimuli.

The 5 min rest interval was selected to allow partial autonomic recovery and stabilization of heart rate and breathing before the subsequent recording. This duration is commonly used in short-term HRV protocols and was considered sufficient given the low-intensity nature of the preceding tasks. All rest periods were standardized across participants to ensure consistency of measurements.

### 2.5. HRV Analysis

We analyzed HRV parameters in both the time and frequency domains, including normalized indices. Data were processed using MATLAB 2017b (MathWorks, Natick, MA, USA) and R v4.0.5.

Time-domain parameters were as follows:

− SDNN: Standard deviation of all normal-to-normal (NN) intervals.
− rMSSD: Root mean square of successive differences between adjacent NN intervals [27].

Spectral domain parameters (absolute values) were as follows:

− TP (total power): representing all frequencies ≤ 0.4 Hz.
− HF (high frequency ms^2^/Hz): frequency regime in the range 0.15–0.4 Hz; marker of parasympathetic activity.
− LF (low frequency ms^2^/Hz) frequency regime in the range 0.04–0.15 Hz; reflects both sympathetic and parasympathetic modulation, predominantly sympathetic.
− VLF (very low frequency ms^2^/Hz): recording frequencies between 0.0033 and 0.04 Hz; often associated with sympathetic activity.
− ULF (ultra- low frequency ms^2^/Hz): frequency regime ≤ 0.0033 Hz; its physiological significance remains uncertain [24,27,28].

Spectral domain parameters (relative values) were as follows:

− nHF/nhf (the relative value of HF): represents the relative portion of the HF band in the TP band. It is calculated according to the following formulas: nHF = HF**/**(TP − VLF) or nhF = HF**/**(HF + LF) [29,30].
− nLF/nlf (the relative value of LF): represents the relative portion of the LF band in the TP band. It is calculated according to the following formulas: nLF = LF**/**(TP − VLF) or nLF = LF**/**(HF + LF) [29,30].
− LF/HF ratio.

The analysis of both time and spectral HRV parameters is important because they reflect the activity of the sympathetic and parasympathetic branches of the ANS. HF activity is interpreted as a surrogate for PNS activity, such as rMSSD; LF reflects changes in both arms of the ANS, primarily sympathetic activity; and increased levels in the VLF area are associated with increased sympathetic activity, according to most authors [29,31,32]. Regarding the ULF band, it is unclear if the primary contributor to ULF power is the sympathetic or the parasympathetic nervous system. LF/HF reflects the global balance between sympathetic/parasympathetic activity [24]. SDNN, influenced by both branches of the ANS (SNS and PNS), correlates strongly with TP, ULF, VLF, and LF spectral power [33]. The normalized coherence parameter (Ncoh) was expressed as a percentage.

The study protocol was designed according to established guidelines for evaluating autonomic responses to orthostatic challenge and emotional stimulation. The sequence of conditions, recording duration (5 min), and controlled environmental settings followed published recommendations for short-term HRV analysis. The protocol was reviewed and approved by the institutional ethics committee before implementation. While no formal external validation was performed, the protocol was pilot-tested for feasibility and applied uniformly to all participants.

### 2.6. Statistical Analysis

We compared the heart rate (HR), time domain HRV parameters, and the frequency domain HRV parameters, either expressed in absolute or relative values, for each session. The analyses were performed using MATLAB (MathWorks, Natick, MA, USA) and R v4.0.5. The graphical displays obtained show variations in depression and anxiety scores according to gender and age.

Linear regression analyses were performed using MATLAB’s “fitlm” function to model relationships between response variables (HR, SDNN, TP, LF, HF, VLF, ULF, rMSSD, Ncoh) and predictor variables (Sex and Age, Sex and Depression Score, or Sex and Anxiety Score). Two-way ANOVA was applied to the table resulting from the linear regression analysis. This provides information about levels of variability within the regression model and forms a basis for tests of significance [34].

Participants were grouped by median age (26–28 years), anxiety score (0–7 vs. 8–21), and depression score (0–7 vs. 8–21). Only subgroups with sufficient sample size were included in the statistical analyses.

## 3. Results

A total of 31 participants were initially enrolled in the study. After visual inspection of the cardiac electrical activity recordings, 24 subjects (17 females and 7 males) were retained for analysis. This visual inspection was performed to avoid distortion in both time and frequency-domain measurements. We excluded those subjects in which noises were recorded, expressed as missed or spurious beats, or those in which the frequency range exceeded flagrantly the interval established in the specialized literature [35].

### 3.1. General HRV Parameters

The general study cohort (GL) included 24 subjects, 17 females (F) and 7 males (M). The mean age of participants was 27.04 years (standard deviation 1.97). The mean score for anxiety was 7.04 (std = 3.97, median = 6, and [IQR] = 4–10), and the mean depression score was 3.13 (std = 2.27, median = 2.5, and [IQR] = 2–4).

According to the HADS classification, two subgroups were formed for each dimension:

Anxiety: A0–7 (score ≤ 7; *n* = 15; 7 males, 8 females) and A8–21 (score ≥ 8; *n* = 9; all females).

Depression: D0–7 (score ≤ 7; *n* = 22; 7 males, 15 females) and D8–21 (score ≥ 8; *n* = 2; both females).

Analyzed a lot of studies that consisted of the GL, as well as the A0-7, A8-21, D0-7, and D8-21 subgroups. Due to the small sample size, subgroup D8-21 was excluded from statistical testing. Parameters evolution with age, anxiety, and depression scores; comparative evolution between sessions for each parameter (Table 1).

The main HRV differences between all sessions are included in the following table.

**Table 1:** The comparative evolution of HRV parameters between sessions.

GL-(24p)	Standing (B) vs. Basal (A)	Positive Image Watching (C) vs. Basal (A)	Negative Image Watching (D) vs. Basal (A)	Negative Image Watching (D) vs. Positive Images Watching (C)
	F M	F M	F M	F M
	*p*-Value	*p*-Value	*p*-Value	*p*-Value
	(Effect-Size ƞ^2^)	(Effect-Size ƞ^2^)	(Effect-Size ƞ^2^)	(Effect-Size ƞ^2^)
HR				
GL	+ +			
	0.0002 *			
	(0.02)			
A0-7	+ +			
	0.0015 *			
	(0.0003)			
D0-7	+ +			
	0.0004 *			
	(0.04)			
A8-21	+			
	0.0325 *			
	(0.26)			
nLF				
GL	+ ++	+ +	+ ++	0 +
	0.0094 *	0.0326 *	0.0091 *	0.0380 *
	0.0053 ^#^		0.0254 ^#^	
	(0.16)	(0.05)	(0.11)	(0.09)
A0-7	0 +		+ ++	0 +
	0.0141 ^#^		0.0495	0.0148 ^#^
	(0.2)		(0.14)	(0.2)
D0-7	+ ++		+ ++	0 +
	0.0192 *		0.0165 *	0.0165 ^#^
	0.0084 ^#^		0.0292 ^#^	
	(0.16)		(0.11)	(0.13)
A8-21				
nlf				
GL	+ ++		+ ++	+ ++
	0.0165 *		0.0044 *	0.0291 ^#^
	0.0072 ^#^		0.0140 ^#^	
	(0.15)		(0.13)	(0.1)
A0-7	+ ++ 0.0150 ^#^ (0.2)		+ ++ 0.0216 * 0.0374 ^#^ (0.15)	+ ++ 0.0189 ^#^ (0.19)
D0-7	+ ++		+ ++	+ ++
	0.0275 *		0.0146 *	0.0084 ^#^
	0.0056 ^#^		0.0119 ^#^	
	(0.17)		(0.14)	(0.16)
A8-21				
nHF				
GL	- - -		- - -	- - -
	0.0455 *		0.0166 *	0.0386 ^#^
	0.0149 ^#^		0.0259 ^#^	
	(0.12)		(0.11)	(0.09)
A0-7	- - - 0.0217 ^#^ (0.18)		- - - 0.0227 * 0.0319 ^#^ (0.16)	- - - 0.0194 ^#^ (0.19)
D0-7	- - -		- - -	- - -
	0.0570 *		0.0400 *	0.0075 ^#^
	0.0095 ^#^		0.0117 ^#^	
	(0.15)		(0.15)	(0.16)
A8-21				
nhf				
GL	- - -		- - -	0 -
	0.0165 *		0.0070 *	0.0212 ^#^
	0.0072 ^#^		0.0091 ^#^	
	(0.15)		(0.14)	(0.11)
A0-7	- - - 0.0150 ^#^ (0.2)		- - - 0.0339 * 0.0201 ^#^ (0.18)	- - - 0.0101 ^#^ (0.22)
D0-7	- - -		- - -	- - -
	0.0275 *		0.0224 *	0.0054 ^#^
	0.0056 ^#^		0.0072 ^#^	
	(0.17)		(0.16)	(0.17)
A8-21				
LF/HF				
GL	+ ++	+ +	+ ++	- +
	0.0227 *	0.0246 *	0.0056 *	0.0434 ^#^
	0.0217 ^#^		0.0091 ^#^	
	(0.11)	(0.05)	(0.14)	(0.09)
A0-7	+ ++	+ ++	+ ++	+ ++
	0.0122 *	0.0488 ^#^	0.0381 *	0.0302 ^#^
	0.0434 ^#^		0.0540 ^#^	
	(0.14)	(0.14)	(0.13)	(0.16)
D0-7	+ ++	+ ++	+ ++	+ ++
	0.0042 *	0.0339 ^#^	0.0160 *	0.0089 ^#^
	0.0062 ^#^		0.0077 ^#^	
	(0.17)	(0.11)	(0.16)	(0.16)
A8-21		+	+	
		0.0514 *	0.0532 *	
		(0.22)	(0.21)	
NCoh				
GL	+ ++	+ +	+ +	
	0.0067 *	0.0201 *	0.0079 *	
	0.0118 ^#^			
	(0.13)	(0.02)	(0.01)	
A0-7	+ ++	+ +	+ +	
	0.0055 *	0.0576 *	0.0041 *	
	0.0041 ^#^			
	(0.27)	(0.12)	(0.03)	
D0-7	+ ++	+ +	+ +	
	0.0137 *	0.0565 *	0.0080 *	
	0.0179 ^#^			
	(0.13)	(0.03)	(0.01)	
A8-21				

+ increase, ++ higher increase, - decrease, - - a higher decrease; # differences between sexes, * differences between sessions, Basal (A); Standing (B); Exposure to positive images (C); Exposure to negative images (D); GL—general study cohort; A0-7—subjects without anxiety (anxiety score = 0–7); D0-7—subjects without depression (depression score = 0–7); A8-21—subjects with anxiety (anxiety score ≥8); HF—high frequency, nLF = LF/TP − VLF, nlf = LF/HF + LF, nHF = HF/TP − VLF, nhf = HF/HF + LF, NCoh—normalized coherence. The effect size was calculated using the ƞ^2^ score (ƞ^2^ < 0.06 represents a small effect size, 0.06 < ƞ^2^ < 0.14 represents a medium effect size, ƞ^2^ > 0.14 represents a large effect size).

#### 3.1.1. Heart Rate

Heart rate (HR) increased significantly only in the orthostatic position (Session B) compared with rest (Session A) (*p* < 0.001). This increase was observed across all subgroups and did not differ by sex, age, anxiety, or depression score.

However, in female participants, HR responses differed by anxiety status when exposed to emotional images (negative or positive content). Thus, in females with anxiety, HR increased during exposure to positive images, but the result is not statistically significant (Session C vs. A; *p* = 0.0535). On the other hand, in females without anxiety, HR increased during exposure to negative images (Session D vs. A; *p* = 0.0222).

When comparing the two emotional sessions directly (Session D vs. C), HR decreased in females with anxiety when exposed to negative images compared to those exposed to positive images, and HR increased in female subjects without anxiety when exposed to negative images compared with positive image exposure (*p* = 0.0403).

#### 3.1.2. Time Domain HRV Parameters

No significant variation in SDNN was observed across sessions or subgroups. SDNN was not associated with sex, age, anxiety, or depression scores.

In contrast, rMSSD showed inverse trends in males and females when exposed to the orthostatic position: it decreased in females and increased in males with higher depression scores. However, females generally presented higher rMSSD values (*p* = 0.0474).

#### 3.1.3. Spectral HRV Parameters

**Figure 1:**
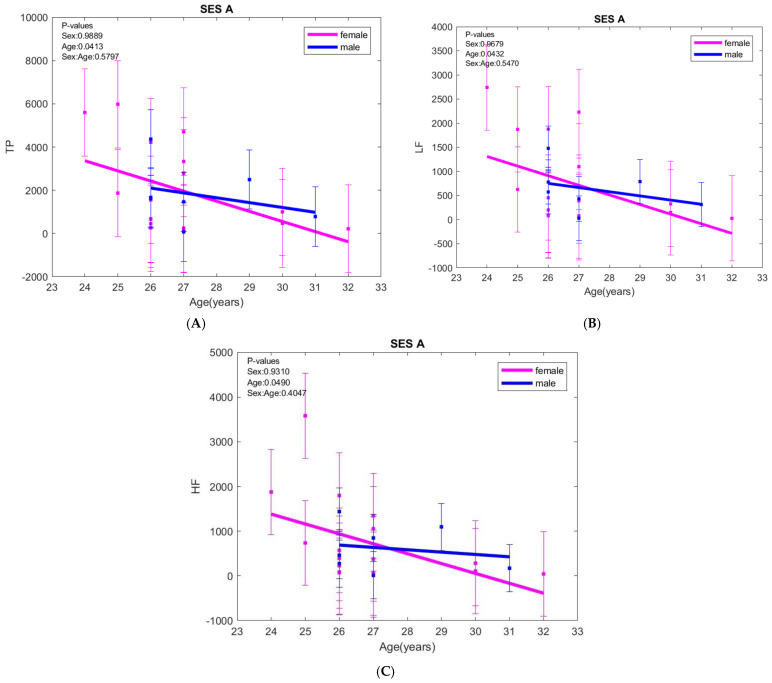
(**A**) Total power (TP), (**B**) low frequency band (LF), and (**C**) high frequency band (HF) variations with age at rest (SES A). The error bars represent ± one standard deviation (SD) from the mean value.The only variation for VLF with age was observed in the B session (standing session), where VLF decreased with age in both sexes (*p* = 0.0573) (Figure 2).

The absolute values of spectral domain HRV parameters at rest, such as TP, LF, and HF, had a similar evolution, decreasing progressively with age in both sexes (*p* = 0.0413, *p* = 0.0432, respectively, *p* = 0.0490) (Figure 1). The same pattern persisted across all sessions, with no sex-related differences.

**Figure 3:**
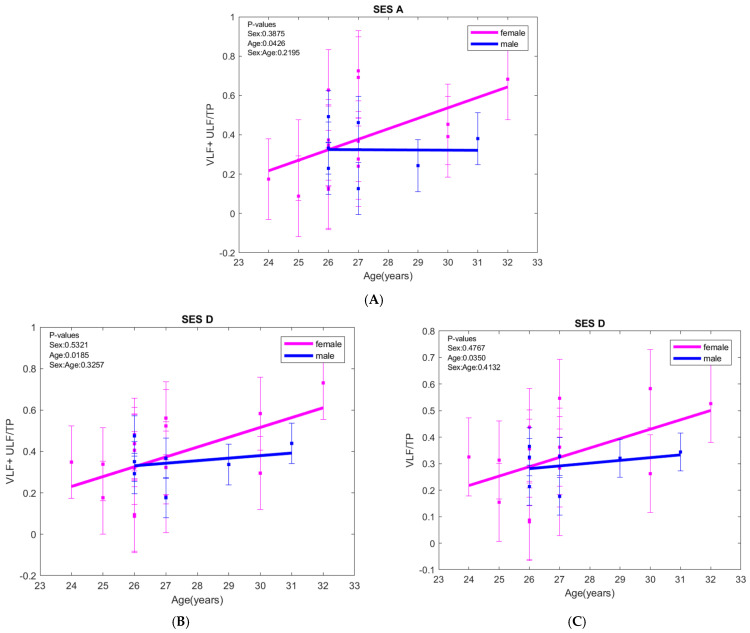
(**A**,**B**) VLF + ULF/TP variations with age at rest (SES A), and when exposed to negative images (SES D); (**C**) VLF/TP variation with age when exposed to negative images (SES D); VLF—very low frequency band, ULF—ultra-low frequency band, TP—total power. The error bars represent ± one standard deviation (SD) from the mean value.

At rest, the ratio (VLF + ULF)/TP increased with age in females but decreased slightly with age in males (*p* = 0.0426). When exposed to negative images (session D), VLF/TP and (VLF + ULF)/TP show the same pattern, increasing with age in both sexes, with VLF/TP (*p* = 0.0350) and (VLF + ULF)/TP (*p* = 0.0185) being statistically significant. No associations were found between VLF/TP and (VLF + ULF)/TP with anxiety or depression scores (Figure 3).

#### 3.1.4. Normalized Frequency Parameters

nLF and nlf

Both nLF and nlf showed their lowest values at rest in GL, irrespective of anxiety or depression score. Both nLF and nlf showed their highest values during standing (Session B). These parameters had no statistically significant changes with age, anxiety, or depression scores. It increased in all sessions compared with rest, predominantly in males.

In the orthostatic session, nLF had a divergent evolution with age in males and females. Thus, nLF decreased with age in males and increased with age in females (*p* = 0.0217). nLF had a parallel evolution regarding anxiety score in both sexes, but higher in males (*p* = 0.0211), increasing with depression score in both sexes, but more markedly in males (*p* = 0.0099). Notably, when analyzing nlf parameter evolution with age and anxiety score, we observed some differences compared with the nLF parameter. Thus, nlf increased with age in both sexes, with higher values in males (*p* = 0.0224), and decreased with anxiety score in males (*p* = 0.0407).

During exposure to negative images (Session D), nlf values were significantly lower in females, showing opposite age-related trends between sexes (*p* = 0.0401).

nLF and nlf tend to increase with anxiety score in both sexes, with higher values in male subjects, relevant only for the nlf parameter (*p* = 0.0458). nlf tended to decrease with depression score in males and to increase in females with depression score, relevant only as differences between sexes (*p* = 0.0280).

nHF and nhf

nHF and nhf exhibit an inverse pattern compared to that of nLF and nlf. Their lowest values were recorded during standing and image exposure, and the highest at rest. No significant differences were observed between anxious and non-anxious females.

**Figure 4:**
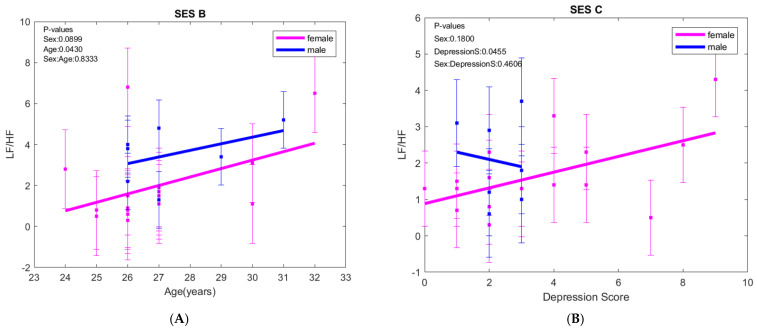
(**A**) LF/HF ratio evolution with age in orthostatic position (SES B) and the (**B**) LF/HF ratio evolution when exposed to positive images (SES C); LF—low frequency, HF—high frequency. The error bars represent ± one standard deviation (SD) from the mean value.

In the orthostatic position, LF/HF increased with age in both sexes. During exposure to positive images (session C), LF/HF increased with depression score in females but decreased in males (Figure 4).

#### 3.1.5. LF/HF Ratio

The LF/HF ratio increased in all sessions (standing, positive image exposure, and negative image watching) compared with rest, particularly in male subjects.

In anxious females, LF/HF was higher during both positive and negative image exposure compared with rest. In males, LF/HF tended to be higher when viewing negative images than when viewing positive images, although this difference did not reach statistical significance.

#### 3.1.6. Normalized Coherence (Ncoh)

Ncoh values were lowest at rest and highest during orthostatic standing. Ncoh increased significantly in all sessions compared with baseline (*p* < 0.05).

Sex differences were observed only in the orthostatic session, in which males exhibited greater Ncoh increases than females (*p* = 0.0011*). During emotional image exposure, Ncoh increased similarly in both sexes. No associations were found between Ncoh and anxiety or depression scores.

### 3.2. Correlations Between HRV Parameters (Figure 5, Figure 6, Figure 7, Figure 8 and Figure 9)

Strong correlations were observed among HRV indices across sessions, as below:

LF/HF and Ncoh: Direct correlations were present in most sessions. For the general cohort, significant associations were found in sessions B (r = 0.755, *p* < 0.001), C (r = 0.438, *p* = 0.032), and D (r = 0.489, *p* = 0.015).

Sex specific correlations: In males, LF/HF correlated with Ncoh only during negative image exposure (session D; r = 0.937, *p* = 0.002). In females, correlations were observed during standing (session B; r = 0.651, *p* = 0.005) and during positive image sessions (session C; r = 0.515, *p* = 0.034).

Anxiety-specific patterns: In non-anxious participants, LF/HF correlated directly with Ncoh during standing (sessions B; r = 0.769, *p* = 0.001) and negative image exposure (session D; r = 0.646, *p* = 0.009). In anxious females, an inverse correlation was observed between LF/HF and Ncoh at rest (session A; r = −0.865, *p* = 0.003).

Inter-session correlations were limited. For the general cohort, Ncoh values correlated directly between standing and positive-image sessions (session B vs. session C; r = 0.424, *p* = 0.039). Differences between sexes were observed: in females, this relationship remained significant (r = 0.598, *p* = 0.011), whereas no inter-session correlations were observed in males. In anxious females, an inverse relationship was found between Ncoh at rest (session A) and Ncoh during positive image exposure (session C) (r = −0.783, *p* = 0.013).

LF/HF was directly correlated with nLF and nlf in nearly all sessions (r > 0.4, *p* < 0.05) and inversely correlated with nHF and nhf (r < −0.4, *p* < 0.05), confirming the reciprocal modulation of sympathetic and parasympathetic components.

**Figure 5:**
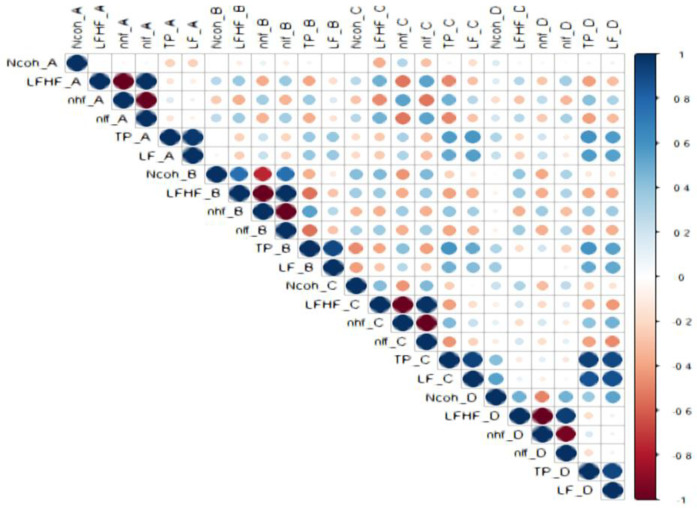
The correlations between HRV parameters in the general study cohort.

**Figure 6:**
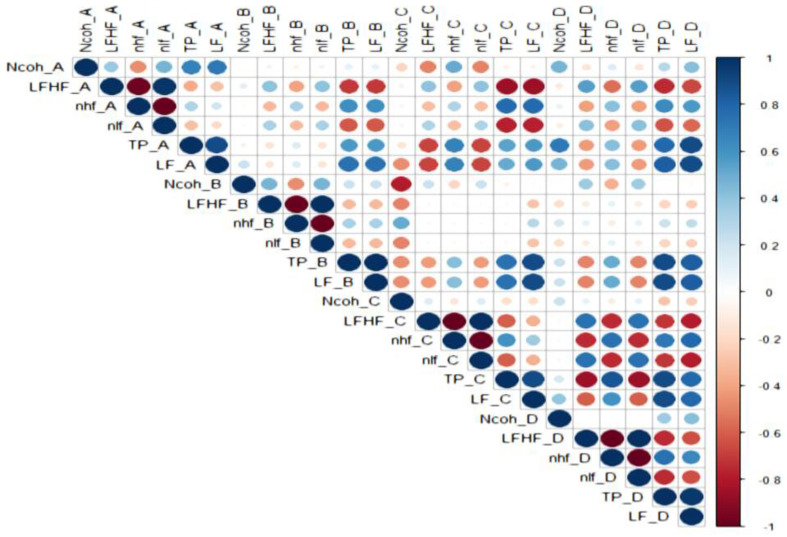
The correlations between HRV parameters in males.

**Figure 7:**
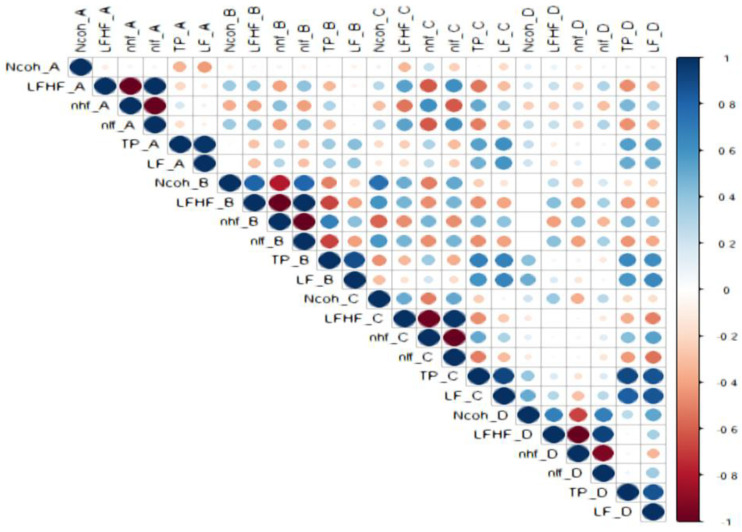
The correlations between HRV parameters in females.

**Figure 8:**
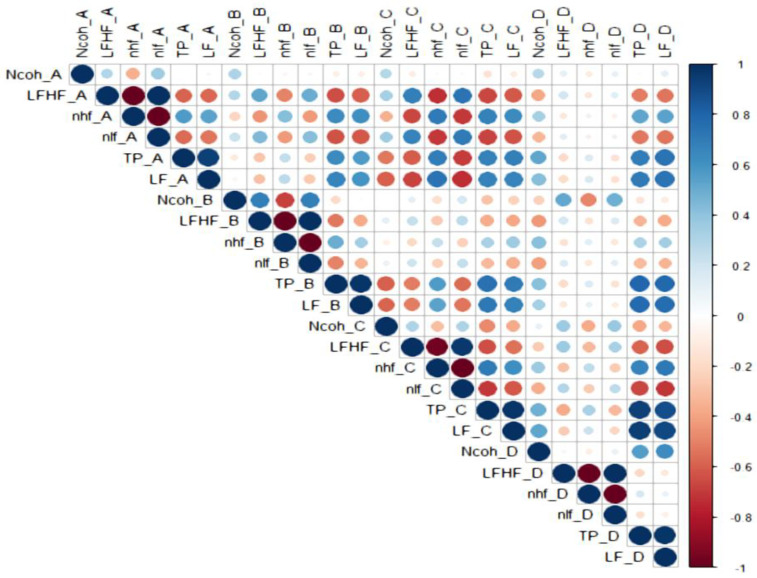
The correlations between HRV parameters in subjects without anxiety.

**Figure 9:**
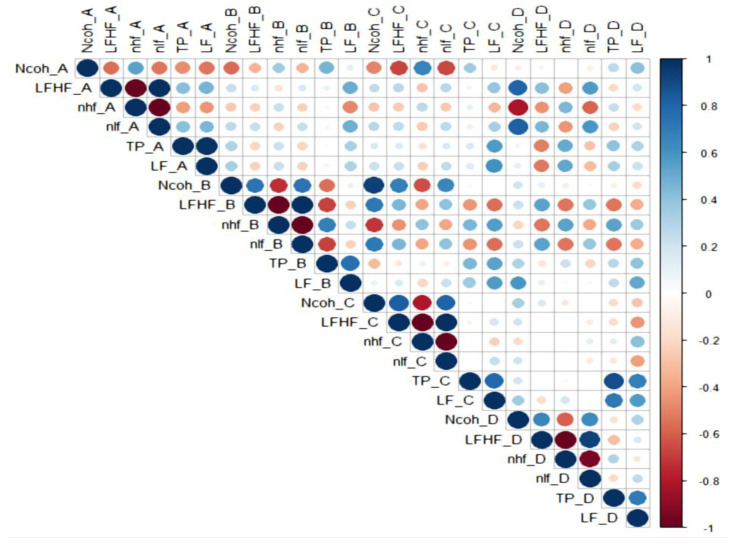
The correlations between HRV parameters in subjects with anxiety.

Legend Figure 5, Figure 6, Figure 7, Figure 8 and Figure 9. Color intensity and circle size are proportional to the correlation coefficients (higher correlation—deeper coloring). Direct correlations are represented by blue circles and inverse correlations by red. Legend color represents correlation coefficients and color. (SDNN—standard deviation of the NN intervals, rMSSD—root mean square of the mean squared differences in successive NN intervals, TP—Total power, HF—High frequency power, LF—Low frequency power, VLF—Very-low frequency power, ULF—Ultra-low frequency power, nLF/nlf—Normalized LF, nHF/nhf—Normalized HF, LF/HF—the ratio of LF power to HF power, NCoh—Normalized coherence, PSD—power spectral density). Session A (Rest): Participant seated comfortably without visual or auditory stimuli; spontaneous breathing; HRV recorded for a duration of 5 min, Session B (Standing): Participant standing upright; HRV recorded for 5 min, Session C (Positive images): Participant seated; viewed 25 positive images for 5 min, Session D (Negative images): Participant seated; viewed 25 negative images for 5 min.

## 4. Discussion

This study investigated autonomic nervous system (ANS) activity, as reflected by heart rate variability (HRV), in young healthy adults exposed to orthostatic stimulation and emotional visual stimuli. We observed that HR increased significantly only in the standing posture, whereas spectral HRV indices—particularly nLF, nlf, LF/HF, and Ncoh—showed marked increases in all active conditions compared with rest. Emotional reactivity seemed to differ according to anxiety status, particularly in females, suggesting a modulatory effect of anxiety on autonomic and emotional responsiveness.

When analyzing the autonomic response to orthostatic posture, the most prominent physiological change was an increase in HR during orthostatic standing. This response was consistent across all participants, independent of age, sex, or depression score. The orthostatic challenge is known to elicit parasympathetic withdrawal and sympathetic activation, resulting in a characteristic shift in HRV toward higher LF and LF/HF values [36]. Our findings align with this pattern, consistent with a robust sympathovagal adaptation to postural change in healthy young adults. Interestingly, females with anxiety exhibited a smaller HR increase during standing than those without anxiety, suggesting a higher basal sympathetic tone and potentially reduced autonomic flexibility. Impaired autonomic responsiveness to orthostatic stress has been linked to anxiety-related dysregulation of vagal control [6,7,8,29].

### 4.1. Autonomic Activity at Rest and During Emotional Stimulation

At rest, nLF, nlf, LF/HF, and Ncoh reached their lowest values, consistent with a predominance of parasympathetic modulation under basal conditions. Exposure to either positive or negative emotional images resulted in increased sympathetic indices (nLF, nlf, LF/HF), while nHF and nhf decreased, indicating vagal withdrawal. These changes were more pronounced in males, suggesting possible sex-related differences in autonomic reactivity to emotional stimuli [29]. An exploratory observation was that there is a possible difference in heart rate responses among female participants according to anxiety status. In anxious females, HR increased when viewing positive images, whereas non-anxious females showed an HR rise during exposure to negative images. This pattern may reflect differences in emotional appraisal or attentional processing associated with anxiety, as previously described in neurovisceral integration models [3,4,15]. However, this interpretation remains hypothetical given the study design.

Regarding sex- and emotion-related variations in HRV parameters, our results revealed significant sex differences across multiple HRV components. Males exhibited higher nLF, nlf, and LF/HF values across all active sessions, whereas in females, preliminary evidence suggests higher nHF and nhf indices, particularly at rest. These findings are consistent with meta-analytic data showing that men generally present higher sympathetic and lower vagal tone than women [29]. The absence of significant HRV differences between positive and negative image exposure supports previous work suggesting that general arousal level, rather than emotional valence, predominantly determines short-term HRV fluctuations [12,18]. Nonetheless, subtle differences emerged in anxiety subgroups: females with anxiety showed inverse correlations between Ncoh at rest and during positive image exposure, possibly reflecting reduced autonomic adaptability.

### 4.2. Normalized Coherence and Autonomic Balance

The Ncoh parameter increased significantly during orthostatic standing and emotional stimulation, paralleling the rise in LF/HF. The strong positive correlation between these indices across most sessions supports the interpretation that greater coherence reflects an enhanced sympathetic–parasympathetic balance and more synchronized cardiac–autonomic coupling [19,37,38].

Sex-specific patterns were observed in these correlations: LF/HF and Ncoh were associated during standing and positive image exposure in females, but only during negative image exposure in males. This divergence may represent distinct autonomic strategies for emotion regulation between sexes. Moreover, in non-anxious participants, LF/HF and Ncoh were positively correlated during orthostatic and negative image sessions. In contrast, in anxious participants, the correlation was inverted at rest, suggesting that anxiety may alter the baseline integration between cardiac coherence and sympathovagal balance.

### 4.3. Spectral Characteristics and Age-Related Effects

Spectral HRV parameters (TP, LF, HF) decreased with age, consistent with previous literature linking reduced HRV to age-related declines in vagal modulation [33]. VLF and (VLF + ULF)/TP showed sex-dependent age-related trends, increasing in females and decreasing in males at rest. These parameters are thought to represent slow regulatory mechanisms related to thermoregulatory and hormonal influences, which may differ between sexes.

Collectively, the clinical implications of our findings indicate that short-term HRV recordings can sensitively capture subtle differences in autonomic regulation across postural and emotional contexts. The results suggest that orthostatic stimulation evokes the most substantial autonomic shift, whereas emotional visual stimuli elicit more nuanced responses that depend on anxiety level and sex.

The observation that anxious females exhibit increased HR during positive image exposure and altered coherence dynamics implies that autonomic flexibility- indexed by HRV- may serve as a physiological marker of emotional dysregulation. These data suggest that HRV may reflect not only autonomic tone but also cognitive–emotional integration, underscoring its value as a biomarker of stress resilience and emotional regulation capacity [1,2,5,19]. The study sample was homogeneous in terms of age and education, reducing variability related to cognitive or occupational differences. Standardized environmental and procedural controls ensured consistent measurement conditions.

The interpretation of frequency-domain HRV indices warrants caution. Although HF power is commonly considered a marker of parasympathetic (vagal) activity and LF power is often interpreted as reflecting predominantly sympathetic modulation, the physiological meaning of these components—particularly LF and the LF/HF ratio—remains debated in the literature, especially in short-term recordings. Several authors have emphasized that LF reflects a complex interaction between sympathetic and parasympathetic influences and may be strongly affected by baroreflex activity and respiratory patterns. Accordingly, in the present study, frequency-domain indices were interpreted as markers of relative autonomic modulation rather than as direct measures of isolated sympathetic or parasympathetic tone.

This study has several limitations that should be considered when interpreting the results.

First, the sample size was relatively small (N = 24), reflecting the limited study duration and the availability of eligible resident doctors during the predefined enrollment period. Although the repeated-measures design increased sensitivity for detecting within-subject autonomic changes, the modest sample size, together with the predominance of female participants, limits the generalizability of sex-specific findings.

Second, the subgroup with elevated depression scores (D8-21) included only two participants and was therefore excluded from inferential statistical analyses. Consequently, conclusions regarding the influence of depressive symptoms on autonomic responses should be interpreted with caution, and future studies with larger and more balanced samples are needed to clarify these associations. Furthermore, a critical limitation of this study is that the subgroup with elevated anxiety scores (HADS-A ≥ 8) consisted exclusively of female participants. As a result, the effects of anxiety cannot be statistically disentangled from sex-related influences within this subgroup. Consequently, findings observed in anxious females should be interpreted with particular caution, as they may reflect the combined or overlapping effects of anxiety and female sex rather than anxiety alone. Future studies including anxious participants of both sexes are required to clarify sex-independent anxiety-related autonomic patterns.

Third, emotional visual stimuli were selected from publicly available online sources rather than from a validated affective-image database. While all participants were exposed to the same standardized set of images under identical conditions, the lack of normative valence and arousal ratings represents a methodological limitation. It may have reduced sensitivity to detect subtle differences between positive and negative emotional stimuli.

Respiratory rate, a known confounder of heart rate variability—particularly in the high-frequency domain—was monitored and controlled using the emWave Pro system throughout all recording sessions. Nevertheless, the absence of independent respiratory signal recording (e.g., respiratory belts or capnography) limits the precision with which respiratory influences could be quantified.

Finally, the study population consisted exclusively of young, healthy medical residents, which ensured cohort homogeneity but restricted the extrapolation of findings to other age groups or clinical populations. Despite these limitations, the controlled experimental conditions and within-subject design support the internal validity of the observed autonomic patterns and provide a foundation for future larger-scale investigations.

However, limitations include the relatively small sample size, especially for high-depression participants, and the restriction to young, healthy adults, which may limit generalizability. Emotional stimuli were limited to static images, which may not elicit the same autonomic responses as dynamic or socially relevant stimuli. Future research should include larger, more diverse cohorts and incorporate multimodal measures such as respiratory monitoring or neuroimaging to elucidate cortical–autonomic interactions.

## 5. Conclusions

In this study, heart rate variability (HRV) analysis revealed distinct autonomic nervous system (ANS) responses to orthostatic and emotional stimuli in young healthy adults. Heart rate increased only during orthostatic challenge, indicating robust sympathetic activation with postural change. In contrast, emotional visual stimuli modulated autonomic balance without altering heart rate, reflecting subtler shifts in sympathovagal dynamics. Spectral and normalized HRV parameters (nLF, nlf, LF/HF, and Ncoh) showed their lowest values at rest and increased significantly during standing and image exposure, particularly in males. Parasympathetic indices (nHF, nhf) exhibited mirrored trends, consistent with reciprocal ANS regulation. Accordingly, interpretations related to sex- and anxiety-associated differences should be viewed as preliminary and not as evidence of independent or causal effects.

Exploratory analyses suggested that sex and anxiety may be associated with differences in autonomic patterns. Females with anxiety displayed higher HR during positive image exposure, while non-anxious females responded more strongly to negative stimuli. Furthermore, coherence (Ncoh) was inversely related between rest and positive-image sessions in anxious females, suggesting altered autonomic adaptability.

The LF/HF ratio correlated positively with Ncoh across all active conditions, supporting their potential joint role as indicators of balanced cardiac–autonomic integration. These preliminary findings suggest that HRV and coherence analysis may provide sensitive markers of physiological and emotional regulation, particularly in experimental settings.

The findings of this study must be interpreted within the context of several important limitations. The sample size was small (N = 24), reflecting the pilot nature of the investigation, and the study population showed a marked sex imbalance. In addition, participants with elevated depression scores were underrepresented and therefore excluded from inferential subgroup analyses, limiting conclusions regarding depressive symptoms. Finally, emotional stimuli were selected from publicly available sources rather than from a validated affective-image database. Consequently, the present results should be regarded as exploratory and hypothesis-generating rather than confirmatory.

## Figures and Tables

**Figure 2:**
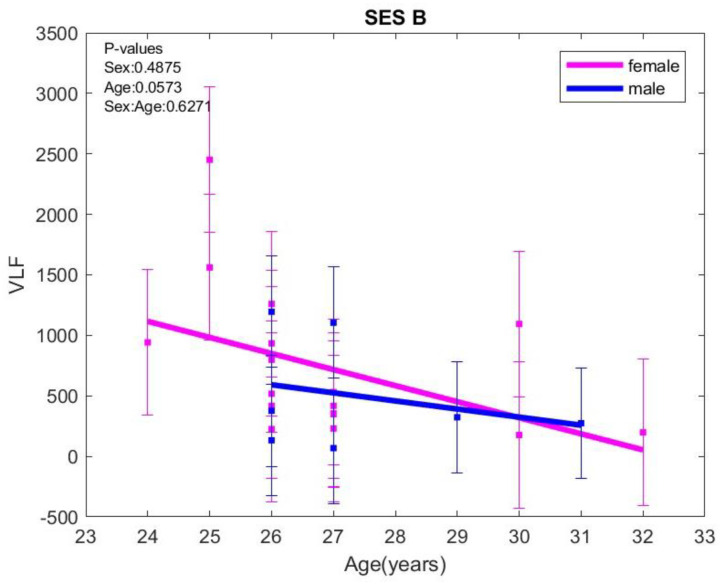
Very low frequency (VLF) variations with age in standing (SES B). The error bars represent ± one standard deviation (SD) from the mean value.

## Data Availability

The data that support the findings of this article are not publicly available, but access can be requested from the corresponding author.

## Abbreviations

The following abbreviations are used in this manuscript:

SDNN	Standard deviation of the NN intervals
rMSSD	Root mean square of the mean squared differences in successive NN intervals
TP	Total power
HF	High-frequency power
LF	Low-frequency power
VLF	Very-low frequency power
ULF	Ultra-low frequency power
nLF/nlf	Normalized LF
nHF/nhf	Normalized HF
LF/HF	The ratio of LF power to HF power
NCoh	Normalized coherence
PSD	Power spectral density
IQR	Interquartile range
